# Application of Ultra-High-Performance Liquid Chromatography Coupled with LTQ-Orbitrap Mass Spectrometry for the Qualitative and Quantitative Analysis of *Polygonum multiflorum* Thumb. and Its Processed Products

**DOI:** 10.3390/molecules21010040

**Published:** 2015-12-26

**Authors:** Teng-Hua Wang, Jing Zhang, Xiao-Hui Qiu, Jun-Qi Bai, You-Heng Gao, Wen Xu

**Affiliations:** 1 Lab of Chinese Materia Medica Preparation, the Second College of Clinic Medicine, Guangzhou University of Chinese Medicine; Guangdong Province Institute of TCM, Guangzhou 510006, China; wangtenghua88@126.com (T.-H.W.); ginniezj@163.com (J.Z.); gxhqxh@medmail.com.cn (X.-H.Q.); baijunqibai@126.com (J.-Q.B.); 2 School of Chinese Materia Medica, Guangzhou University of Chinese Medicine, Guangzhou 510006, China

**Keywords:** *Polygonum multiflorum*, LTQ-Orbitrap, qualitative and quantitative

## Abstract

In order to quickly and simultaneously obtain the chemical profiles and control the quality of the root of *Polygonum multiflorum* Thumb. and its processed form, a rapid qualitative and quantitative method, using ultra-high-performance liquid chromatography coupled with electrospray ionization-linear ion trap-Orbitrap hybrid mass spectrometry (UHPLC-LTQ-Orbitrap MS^n^) has been developed. The analysis was performed within 10 min on an AcQuity UPLC™ BEH C_18_ column with a gradient elution of 0.1% formic acid-acetonitrile at flow rate of 400 μL/min. According to the fragmentation mechanism and high resolution MS^n^ data, a diagnostic ion searching strategy was used for rapid and tentative identification of main phenolic components and 23 compounds were simultaneously identified or tentatively characterized. The difference in chemical profiles between *P. multiflorum* and its processed preparation were observed by comparing the ions abundances of main constituents in the MS spectra and significant changes of eight metabolite biomarkers were detected in the *P. multiflorum* samples and their preparations. In addition, four of the representative phenols, namely gallic acid, *trans*-2,3,5,4′-tetra-hydroxystilbene-2-*O*-β-d-glucopyranoside, emodin and emodin-8-*O-*β-d-glucopyranoside were quantified by the validated UHPLC-MS/MS method. These phenols are considered to be major bioactive constituents in *P. multiflorum*, and are generally regarded as the index for quality assessment of this herb. The method was successfully used to quantify 10 batches of *P. multiflorum* and 10 batches of processed *P.* *multiflorum*. The results demonstrated that the method is simple, rapid, and suitable for the discrimination and quality control of this traditional Chinese herb.

## 1. Introduction

The root of *Polygonum multiflorum* Thumb. (*Fallopia multiflora*), well known as He-shou-wu in China, has been widely used as a tonic and purgative in traditional Chinese medicine for thousands of years [1]. Previous phytochemical studies revealed that the constituents of *P. multiflorum* are mainly two kinds of active constituents—anthraquinones and stilbenes—as well as other compounds such as flavonoids, tannins and phospholipids [2,3,4]. Recently, four novel stilbene derivatives, named polygonumosides A–D, were isolated from the processed root of *P. multiflorum* [5].

The anthraquinones, such as emodin (EM) and emodin-8-*O*-β-d-glucopyranoside (EMG), provide immunomodulating, anti-inflammatory, anticancer, antimutation, antibacterial, gastrointestinal smooth muscle prokinetic actions, dose-dependent protection against myocardial ischemia-reperfusion injury and cerebral ischemia-induced infarct volume reduction biological effects [6,7,8,9]. Some research supports the notion that the stilbenes in PM possess anti-inflammatory [10,11,12], antioxidant activity [13,14,15,16], anti-hyperlipidaemia [17], anti-melanogenic activity, prophylactic and therapeutic activity against Alzheimer’s disease and Parkinson’s disease [18,19,20], free radical scavenging activity [21], and hair growth properties [22,23,24,25]. According to Chinese medicine theory, the crude *P. multiflorum* (CPM) should be processed before use, involving steaming the crude roots with or without black soybean extract (namely *Paozhi*), which could reduce the laxative effects and strengthen the tonic effects [26]. Both the crude *P. multiflorum* (CPM) and processed *P. multiflorum* (PPM) have been officially listed in the successive editions of the Chinese Pharmacopoeia.

As a consequence of the potential medicinal value of PM, several methods have been published for identification and/or quantification of its chemical components, such as high performance liquid chromatography coupled with photodiode array (HPLC-PDA) detection [27], capillary zone electrophoresis (CZE) [28], and HPLC-DAD coupled with electrospray ionization tandem mass spectrometry (ESI-MS^n^) [29,30]. The present method of using several ingredients in evaluating PM preparations cannot reflect the overall changes of chemical composition that occur during the course of processing. Any new method should overcome this problem by using multi-target ingredient determination and fingerprinting analysis technologies simultaneously, which could control the preparation process and product quality precisely, and ensure the stability and effectiveness of products.

In our previous work, the fragmentation pathways of typical constituents and chemical profiles of PM have been studied by an on-line UHPLC-ESI-linear ion trap-Orbitrap hybrid mass spectrometry (LTQ-Orbitrap) method [31,32]. UHPLC coupled with the high resolution tandem mass spectrometric techniques have been proven to be a powerful tool for the rapid identification of unknown components in botanical extracts. In this paper, a simple and rapid method for the comprehensive qualitative and quantitative analysis of the major constituents was successfully developed for quality evaluation of CPM and PPM. Up to now, this is the first report on the simultaneous identification and determination of multiple components in botanical herb products by an UHPLC-LTQ-Orbitrap MS^n^ technique.

## 2. Results and Discussion

### 2.1. Optimization of the LC-MS Conditions

**Figure 1:**
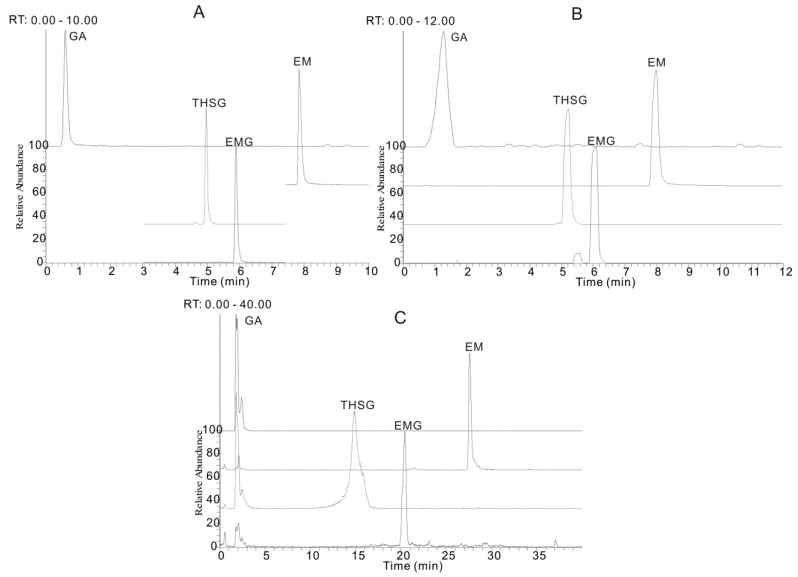
The extracted ion chromatograms (EIC) of gallic acid (GA), *trans*-2,3,5,4′-tetrahydroxystilbene-2-*O*-β-d-glucopyranoside (THSG), emodin-8-*O*-β-d-glucopyranoside (EMG) and emodin (EM) with three different reversed-phase columns. (**A**) AcQuity UPLC™ BEH C_18_ column (2.1 mm × 50 mm, 1.7 μm); (**B**) Kinetex XB C_18_ (2.1 mm × 100 mm, 1.7 μm) and (**C**) Hypersil C_18_ (2.1 mm × 100 mm, 5 μm). The mobile phases consisted of acetonitrile (a) and water containing 0.1% formic acid (b), with the following elution gradient program: (**A**) used the optimized mobile phase and gradient (see Section 3.2); (**B**) 13% a (0 min), 35% a (3.5 min), 90% a (7.5 min), 95% a (8.5 min) and 95% a (12 min); (**C**). 13% a (0 min), 35% a (12 min), 90% a (32 min), 95% a (35 min) and 95% a (40 min).

The chromatographic conditions, such as the chromatographic column and mobile phase, were optimized to achieve the best separation efficiency. Three reversed-phase chromatographic columns, including a Hypersil C_18_ (2.1 mm × 100 mm, 5 μm, Thermo Fisher Scientific, Carlsbad, CA, USA), a Kinetex XB C_18_ (2.1 mm × 100 mm, 1.7 μm, Phenomenex, Torrance, WA, USA) and an AcQuity UPLC™ BEH C_18_ column (2.1 mm × 50 mm, 1.7 μm, Waters, Milford, MA, USA), were selected to test the separation ability of the four investigated compounds. It was shown that an UHPLC system with a 1.7 μm small particle size column had more powerful separation ability with a higher peak resolution and the total analysis time was less than 10 min, which was approximately fourfold faster than that for a conventional column packed with 5 μm particles (Figure 1). Thus the AcQuity UPLC™ BEH C_18_ column (2.1 mm × 50 mm, 1.7 μm) was proved to be the best in this application.

**Figure 2:**
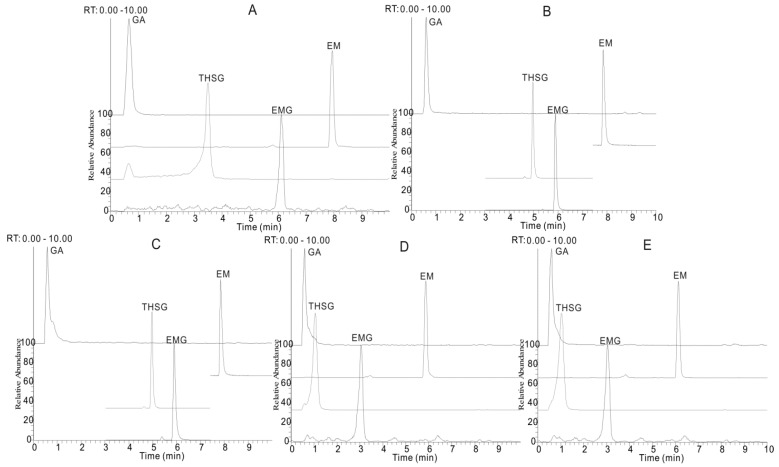
The extracted ion chromatograms (EIC) of gallic acid (GA), *trans*-2,3,5,4′-tetra-hydroxystilbene-2-*O*-β-d-glucopyranoside (THSG), emodin-8-*O*-β-d-glucopyranoside (EMG) and emodin (EM) under five different chromatographic conditions: (**A**) methanol/water system containing 0.1% formic acid; acetonitrile/water system; (**B**) containing 0.1% formic acid; (**C**) containing 0.1% acetic acid; (**D**) containing 0.1% ammonium acetate and (**E**) containing 0.1% ammonium formate.

The experimental results showed that an acetonitrile-H_2_O mobile phase separated the target compounds more effectively (Figure 2B) than a methanol-H_2_O system (Figure 2A). Thus acetonitrile was selected as the organic phase, so as to obtain the optimal separation of adjacent peaks and to avoid peak tailing. Formic acid (Figure 2B), acetic acid (Figure 2C), ammonium acetate (Figure 2D) and ammonium formate (Figure 2E) were added to the mobile phase, respectively, to achieve high MS sensitivity and restrain peak tailing. Formic acid was screened as an effective mobile phase additive with which improvements of peak shape, peak width and decreased ion suppression effects could be directly and efficiently obtained, compared to others.

### 2.2. Sample Extraction Optimization

In order to optimize the sample extraction conditions, the factors affecting the extraction efficiency of the four main compounds, including extraction solvents, solid-liquid ratio and extraction times, were studied. In our research, the extraction efficiency was evaluated by using a single factor method and choosing the extraction yield (extraction yield = the extract weight × the content of target analyte/the weight of crude drug × 100%) as the index. The results suggested that single-solvent system (pure methanol) was superior to a binary solvent system in the extraction of all four compounds (Table 1). The extraction yield was as high as 5.27% (summation of the four test components) as a maximum value with methanol at 100%. Furthermore, the extraction yield increased by 75.9% (summation of the four test components) when the amount of liquid was increased from 10 to 25 mL (*i.e*., solid to liquid ratio from 1:50 to 1:125). When the liquid amount was 50 mL (solid to liquid ratio 1:250), the average extraction yield and extraction yield of gallic acid showed no obvious increase. Thus the solid to liquid ratio of 1:125 was recommended. It was also found that when the extraction time was within 30 min, the extraction yield increased significantly with extraction time. The total extraction yield was 5.85% and the highest yield was reached at an extraction time of 30 min. After that, the yield decreased slightly with time. According to the above results, the optimum extraction conditions were: solid-liquid ratio 1:125 and extraction with 25 mL of 100% methanol at reflux for 30 min.

**Table 1:** Effects of methanol concentration, solid-liquid ratio and extraction time on extraction yield ^a^.

Compound	Methanol Concentration (%)			Solid-Liquid Ratio			Extraction Time (min)		
	50	70	100	1:50	1:125	1:250	15	30	60
Gallic acid	0.46	0.52	0.67	0.69	0.88	0.89	0.26	0.77	0.73
Emodin	1.25	1.42	1.55	1.23	1.75	1.77	0.37	1.88	1.68
*trans*-2,3,5,4′-Tetrahydroxy-stilbene-2-*O-*β-d-glucopyranoside	1.26	1.32	1.61	1.18	1.93	1.69	0.79	1.78	1.86
Emodin-8-*O-*β-d-glucopyranoside	0.86	1.46	1.44	0.43	1.65	1.44	0.73	1.42	1.22

^a^ extraction yield (%) = the extract weight × the content of target analyte/the weight of crude drug × 100%.

### 2.3. Tentative Identification of the Major Compounds

**Figure 3:**
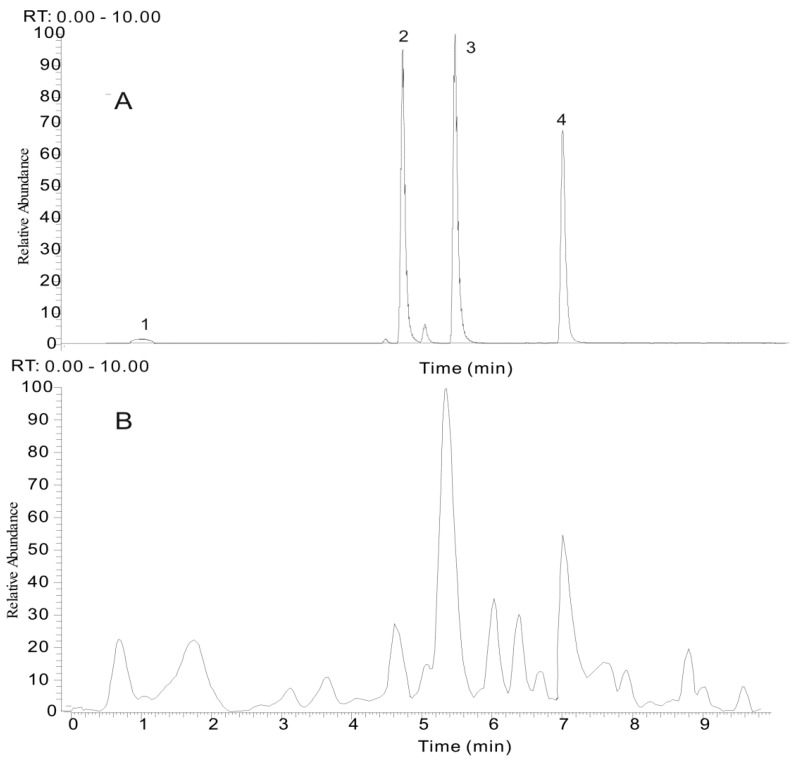
The selected reaction monitoring (SRM) chromatogram of four compounds (**A**) and the total ion chromatogram (TIC) of typical *P. multiflorum* sample (**B**). (1) gallic acid (GA), (2) *trans-*2,3,5,4′-tetrahydroxystilbene-2-*O-*β-d-glucopyranoside (THSG), (3) emodin-8-*O-*β-d-glucopyranoside (EMG) and (4) emodin (EM).

The four reference compounds (gallic acid, EM, THSG and EMG) were initially analyzed to obtain the corresponding retention times and characteristic fragmentation pathway data (Figure 3). The precise quasi-molecular and fragment ions were determined within a reasonable degree of measurement error using the Orbitrap instrument (the mass error is less than 2 ppm in most cases). Other clues such as potential elemental composition, degree of unsaturation and fractional isotope abundance of the compounds were also utilized for structural conformation. In our previous work, the fragmentation rules and diagnostic fragment ions of anthraquinones, stilbenes, tannins and naphthalenes have been investigated, which provided useful information for the elucidation of the chemical structures of the prescription components. As for the untargeted compounds, the characteristic diagnostic ions could be used to filter and classify them into a particular chemical family.

Twenty five compounds, including anthraquinones, stilbenes, tannins and naphthalenes were primarily identified in both of the extracts of CPM and PPM, and three compounds were only detected in PPM, which were probably new components produced during the steaming process (Table 2).

**Table 2:** Identification of the chemical constituents in methanol extracts of CPM and PPM by UHPLC-LTQ-Orbitrap MS.

No.	*t*_R_ (min)	Precursor ions [M − H]	Formula	Mass Error (ppm)	MS^n^	Identification
1	0.89	341.1078	C_12_H_21_O_11_	0.18	MS^2^[377]: 341 (100), 215 (15)	Sucrose *
		377.0843 [M + Cl]			MS^3^: 179 (100), 161 (23), 143 (22), 113 (17)	
2	1.12	179.0556 225.0609 [M + HCOO]	C_6_H_11_O_6_	3.38	MS^2^: 161 (100), 143 (90), 119 (44), 113 (40), 89 (40)	Glucose *
3	2.17	169.0136	C_7_H_5_O_5_	2.66	MS^2^: 125 (100)	gallic acid *
					MS^3^: 81	
4	4.24	577.1330	C_30_H_25_O_12_	−1.8	MS^2^: 425 (100), 407 (48), 457 (20), 471 (17), 289 (10)	procyanidin B
					MS^3^: 407 (100)	
5	4.34	289.0708	C_15_H_13_O_6_	0.47	MS^2^: 245 (100), 205 (42), 179 (19)	epicatechin/catechin
					MS^3^: 203 (100), 227 (23), 187 (22), 161 (20)	
6 ^#^	4.48	531.1488	C_26_H_27_O_12_	−2.2	MS^2^: 369 (100), 351 (29), 405 (21), 243 (18)	unknown
					MS^3^: 351 (100)	
7 ^#^	4.63	549.1594	C_26_H_29_O_13_	−0.97	MS^2^: 387 (100), 459 (73), 531 (22), 297 (16)	unknown
8	4.82	577.1330	C_30_H_25_O_12_	−1.53	MS^2^: 425 (100), 407 (48), 457 (20), 471 (17), 289 (10)	procyanidin B
					MS^3^: 407 (100)	
9 ^#^	4.98	421.1123	C_20_H_21_O_10_	−1.38	MS^2^: 259 (100)	6-methoxyl-2-acetyl-3-methyljuglone-8-*O*-glu
					MS^3^: 259 (100), 331 (50), 128 (20)	
10	5.02	613.1751 [M + HCOO]	C_27_H_33_O_16_	−1.21	MS^2^: 405 (100), 567 (36)	tetrahydroxystilbene-*O*-di-glu
					MS^3^: 243 (100)	
11	5.37	405.1177	C_20_H_21_O_9_	−0.74	MS^2^: 243 (100)	THSG *
		811.2428 [2M − H]			MS^3^: 225 (100), 149 (79), 137 (73), 215 (70), 173 (36)	
12	5.41	557.1286	C_27_H_25_O_1__3_	−0.66	MS^2^: 313 (100), 243 (30), 405 (20), 169 (5)	tetrahydroxystilbene-*O*-(galloyl)-glu
					MS^3^: 169 (100), 125 (20), 151 (20), 295 (17)	
13	5.71	557.1285	C_27_H_25_O_13_	−0.82	MS^2^: 313 (100), 243 (80), 405 (70), 169 (10)	tetrahydroxystilbene-*O*-(galloyl)-glu
					MS^3^: 169 (100), 125 (20), 151 (20), 295 (17)	
14	5.72	431.0970	C_21_H_19_O_10_	−0.63	MS^2^: 269 (100)	emodin-1-*O*-glu
					MS^3^: 225 (100), 241 (21), 181 (4)	
15	5.75	567.1488	C_29_H_27_O_12_	−1.59	MS^2^: 243 (100)	tetrahydroxystilbene-*O*-(caffeoyl)-glu
					MS^3^: 225 (100), 215 (72), 149 (67)	
16	5.90	551.1543	C_29_H_27_O_11_	−0.89	MS^2^: 405 (100), 243 (31)	tetrahydroxystilbene-2-*O*-(coumaroyl)-glu
17	5.92	447.0919	C_21_H_19_O_11_	−0.64	MS^2^: 303 (100), 285 (100)	citreorosein-*O*-glu
					MS^3^: 285 (100), 177 (11), 125 (8)	
18	6.02	407.1334	C_20_H_23_O_9_	−0.64	MS^2^: 245 (100)	torachrysone-*O*-glu
					MS^3^: 230 (100)	
19	6.11	431.0973	C_21_H_19_O_10_	0.11	MS^2^: 269 (100)	emodin-8-*O*-glu *
					MS^3^: 225 (100), 241 (21), 197 (5)	
20	6.26	517.0978	C_24_H_21_O_13_	0.26	MS^2^: 473 (100), 431 (10)	emodin-*O*-(malonyl)-glu
					MS^3^: 269 (100), 311 (12), 225 (5)	
21	6.38	445.1127	C_22_H_21_O_10_	−0.22	MS^2^: 283 (100), 445 (42)	physcion-8-*O*-glu
		491.1182 [M + HCOO]			MS^3^: 240 (100), 268 (36)	
22	6.42	313.0345	C_16_H_9_O_7_	0.96	MS^2^: 269	carboxyl emodin
					MS^3^: 225, 241, 197	
23	7.21	269.0447	C_15_H_9_O_5_	0.33	MS^2^: 225, 241	Emodin *
24	7.63	269.0446	C_15_H_9_O_5_	0.17	MS^2^: 225, 241, 254	aloe-emodin
25	8.30	283.0605	C_16_H_11_O_5_	1.41	MS^2^: 240	physcion

* Compared with standard compouds; ^#^ Detected only in methanol extracts of PPM.

#### 2.3.1. Identification of the Main Stilbene Glycosides

**Figure 4:**
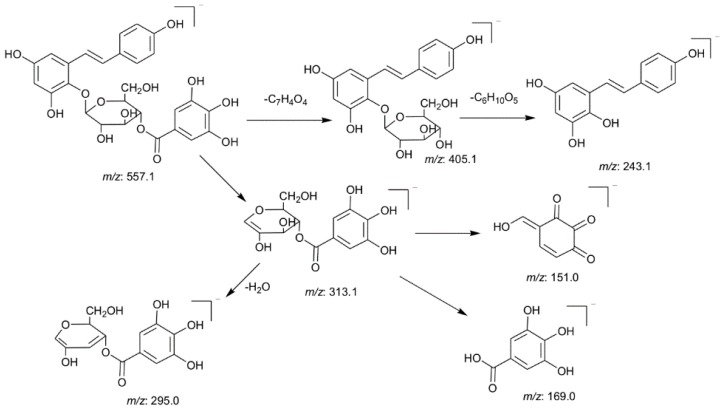
The proposed fragmentation pathways of peaks 12 and 13.

Most of the stilbene glycosides in PM showed common fragmentation pathways and two diagnostic fragment ions, 405.1177 (C_20_H_21_O_9_) and 243.0661 (C_14_H_11_O_4_), were used for rapidly extracting unknown tetrahydroxystilbene glucosides. For example, peaks 12 and 13 exhibited a molecular ion at *m*/*z* 557.1286 (C_27_H_25_O_13_) in the negative ion mode. It produced a base peak at 313.0548 (C_13_H_13_O_9_) and two prominent fragment ions at *m*/*z* 405.1164 (C_20_H_21_O_9_) and 243.0649 (C_14_H_11_O_4_) in the MS^2^ spectra, indicating it was a tetrahydroxystilbene derivative. Then, the *m/z* 313 ion generated the characteristic ions of gallic acid, such as *m*/*z* 169 and 125. Thus, peaks 12 and 13 were tentatively identified as tetrahydroxystilbene-*O*-(galloyl)-glucoside and the proposed fragmentation pathways are shown in Figure 4.

#### 2.3.2. Identification of the Main Anthraquinones

Most of the anthraquinones in PM are emodin and physcion derivatives and they generated typical adducts [M + HCOO]^−^ and/or [M − H]^−^ with high intensity in negative ion mode. Diagnostic ions at *m/z* 269.0447, 225.0544 and 241.0492 could be used for rapid extraction and identification of emodin derivatives. For physcion derivatives, two odd-electron ions (OE^−^) at *m*/*z* 240 and 268 were produced from the simultaneous loss of free radicals on the side chains, which could be used for the identification of such derivatives.

For example, peak 20 gave a [M − H]^−^ ion at *m*/*z* 517.0978 (C_24_H_21_O_13_) and a prominent fragment ion at *m*/*z* 473.1063 (C_23_H_21_O_11_), as well as a characteristic ion at *m*/*z* 431.0960 (C_21_H_19_O_10_). Additional fragmentation yielded diagnostic ions at *m*/*z* 269 and 225 in the MS^3^ spectra. High resolution MS detection indicated it was malonyl-substituted glycoside derivative, which was tentatively identified as emodin-*O*-(malonyl)-glucoside.

### 2.4. Changes of the Relative Intensity of the Main Chemical Components

**Figure 5:**
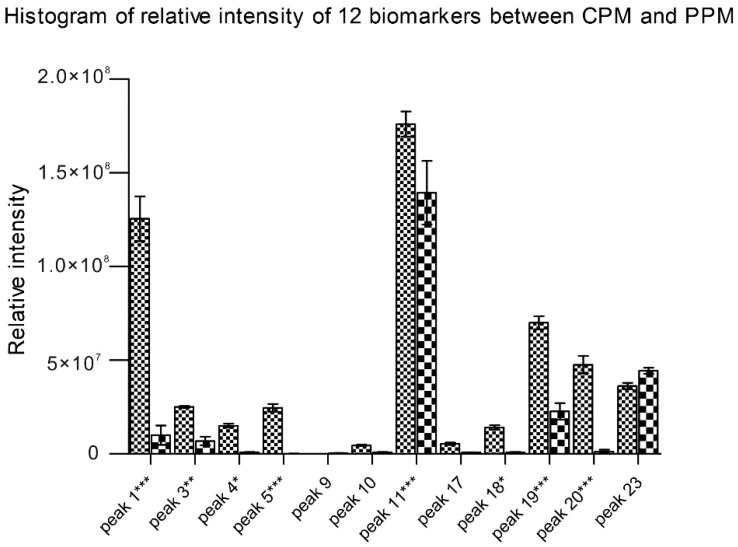
Relative intensity of the extracted ion chromatograms (EIC; mean ± SD) of 12 biomarkers between PM and its processed products. The peak names were originated from Table 2. Statistics are based on three independent experiments for each sample. * *p* < 0.05, ** *p* < 0.01 and *** *p* < 0.001.

Statistical analysis based on the main chemical metabolic components clearly showed differences among 10 batches of CPM and 10 batches of PPM by two-way ANOVA and Bonferroni correction. In examining the relative intensity of the 12 main chemical components in more detail, a histogram of compounds revealed the chemical profiles differ between PM and its processed products. In the present study, obvious differences existed in the compounds sucrose, gallic acid, procyanidin B, catechin, THSG, torachrysone-*O*-glu, emodin-8-*O*-glu and emodin-*O*-(malonyl)-glu between the 10 batches of CPM and 10 batches of PPM (Figure 5). The contents of those compounds were destroyed may due to long-time steaming [33,34].

### 2.5. Quantification of Four Major Phenolic Components of the UHPLC-MS/MS System

A rapid method was developed to quantify four representative phenolic compounds (galic acid, EM, THSG and EMG) in 10 batches of CPM and 10 batches of PPM that were collected from different regions in China (Supplementary Data, Table S1). Since the four investigated bioactive compounds vary greatly in polarity, it is difficult to accomplish a satisfactory separation using a conventional HPLC-UV method. The UHPLC-ESI-MS/MS method has demonstrated higher selectivity, sensitivity and shorter running times, thus it was utilized for rapid determination of these biomarkers. The UHPLC conditions and spectrometric parameters transition were optimized as described in Section 3.2. (Figure 3).

#### 2.5.1. Calibration Curves, LOD and LOQ

A methanol stock solution containing all the reference standards was prepared and diluted to an appropriate concentration range for the construction of calibration curves. At least six concentrations of the solution were analyzed in triplicate, and then the calibration curves were constructed from peak areas of the reference standards *vs.* their concentrations. The high correlation coefficient values (r^2^ > 0.9916) indicated good linearity between their peak areas (y) and investigated compound concentration (x, μg/mL) in relatively wide concentration ranges (Table 3).

The limits of detection (LOD) and the limits of quantification (LOQ) under the chromatographic conditions were determined by injecting a series of standard solutions until the signal-to-noise ratios (S/N) for each compound was 3 for LOD and 10 for LOQ (Table 3).

**Table 3:** Calibration curves, LOD and LDQ of the investigated compounds.

Analytes	Linear Regression Data Regression Equation	Test Range (μg/mL)	r^2^	LOD ^a^ (ng/mL)	LOQ ^b^ (ng/mL)
Gallic acid	y = 3598.5x + 205.82	0.03–4.00	0.9997	0.03	0.10
Emodin	y = 25639x + 2283.1	0.01–1.40	0.9916	0.01	0.02
*trans*-2,3,5,4′-Tetrahydroxy-stilbene-2-*O*-β-d-gluco-pyranoside	y = 652.2x − 5013.2	1.15–105.00	0.9997	0.01	0.02
Emodin-8-*O-*β-d-gluco-pyranoside	y = 7690.7x + 14652	0.29–114.00	0.9998	0.01	0.06

^a^ LOD refers to the limit of detection, *s*/*n* = 3; ^b^ LOQ refers to the limit of quantification, *s*/*n* = 10.

#### 2.5.2. Precision

The precision of the developed assay was evaluated by analyzing the mixed standard solution at three different concentration levels (high, middle and low) (Table 4). The experiment was repeated six times within one day to determine intra-day precision, while for inter-day variability test, the solution was examined in triplicate for consecutive 3 day. Relative standard deviation (RSD) for each of the marker compounds was calculated respectively, and it was no more than 3.27%.

**Table 4:** Intra- and inter-day variability for the assay of four components.

Compound	Intra-Day (*n* = 6)		Inter-Day (*n* = 3)	
	Mean (μg/mL)	RSD ^a^ (%)	Mean (μg/mL)	RSD (%)
Gallic acid	0.01	1.27	0.07	3.27
	0.25	1.15	0.25	1.62
	1.00	0.92	0.97	2.74
Emodin	0.00	1.90	0.04	2.14
	0.34	1.67	0.34	1.87
	2.56	1.48	2.50	2.39
*trans*-2,3,5,4′-Tetrahydroxystilbene-2-*O*-β-d-glucopyranoside	2.17	1.45	2.08	1.68
	10.10	1.23	10.12	2.71
	107.67	0.77	106.33	1.65
Emodin-8-*O-*β*-*d-glucopyranoside	0.37	1.14	0.37	2.45
	1.52	1.49	1.50	1.49
	67.88	1.30	68.13	2.57

^a^ RSD (%) = (SD/mean) × 100%.

#### 2.5.3. Repeatability, Stability, Specificity and Selectivity

The repeatability of this method was determined by analyzing six samples from the same batch (Sample A6) using the same preparation procedure described in Section 3.3 of the paper. The RSD values of the four component contents were all less than 3.81% (Table 5). The stability was tested by analysis of the same sample solution (Sample A6) which was at room temperature at different times for 24 h. The RSD values of the four analytes were less than 2.01%, which suggested that it was feasible to analyze samples within 24 h (Table 5).

**Table 5:** Repeatability and stability of the four analytes, expressed as RSD (%).

Compound	Repeatability (*n* = 6)		Stability (*n* = 6)	
	Mean (μg/mL)	RSD ^a^ (%)	Mean (μg/mL)	RSD (%)
Gallic acid	0.33	3.81	0.34	2.01
Emodin	1.04	2.53	1.02	1.17
*trans*-2,3,5,4′-Tetrahydroxystilbene-2-*O*-β-d-glucopyranoside	27.34	1.85	27.83	1.49
Emodin-8-*O-*β-d-glucopyranoside	0.83	2.98	0.80	1.37

^a^ RSD (%) = (SD/mean) × 100%.

#### 2.5.4. Accuracy

Recovery test was used to evaluate the accuracy of this method. A known amount (low, medium and high) of each standard solution was spiked into known amounts of CPM samples (simple A6), and then extracted according to the section of sample pretreatment. The recoveries were counted by the formula: recovery (%) = concentration found/original concentration × 100%. The recoveries of analytes varied from 97.34% to 100.75% (Table 6). The result indicated the reliability and accuracy for the quantitative determination of the constituents.

**Table 6:** Recoveries of the four determined constituents.

Compound	Amount (μg/mL)		Recovery ^a^ (%)	RSD ^b^ (%)
	Spiked	Found		
Gallic acid	0.26	0.26 ± 0.00	98.39	1.19
	0.33	0.32 ± 0.01	98.87	1.81
	0.39	0.39 ± 0.00	98.54	0.04
Emodin	0.90	0.91 ± 0.00	100.75	0.06
	1.12	1.13 ± 0.02	100.53	1.43
	1.35	1.33 ± 0.01	98.73	1.41
*trans*-2,3,5,4′-Tetrahydroxystilbene-2-*O*-β-d-glucopyranoside	23.46	22.83 ± 0.14	97.34	0.63
	29.32	29.43 ± 0.37	100.37	1.26
	35.19	34.54 ± 0.40	98.17	1.18
Emodin-8-*O*-β-d-glucopyranoside	7.35	7.17 ± 0.06	97.56	0.77
	9.19	9.16 ± 0.13	99.75	1.46
	11.03	11.07 ± 0.05	100.40	0.43

^a^ Recovery (%) = (detected amount − original amount)/spiked amount × 100%. ^b^ RSD (%) = (SD/mean) × 100%.

#### 2.5.5. Sample Analysis

Twenty batches of *P. multiflorum* acquired from different regions of China were determined using the described method. This work disclosed that the variation between different locations and processing methods of this traditional Chinese medicine was obvious. Such variations may presumably be attributed to differences in the cultivation conditions (Table 7).

**Table 7:** Contents of four components in 20 samples.

No.	Content (mg/g) (*n* = 3)			
	Gallic Acid (GA)	Emodin (EM)	2,3,5,4′-Tetrahydroxystilbene-2-*O*-β-d-glucoside (THSG)	Emodin-8-*O*-β-d-glucoside (EMG)
A1	0.63 ± 0.01	1.01 ± 0.02	34.43 ± 0.59	21.11 ± 0.34
A2	0.44 ± 0.05	0.23 ± 0.00	4.53 ± 0.15	1.32 ± 0.05
A3	0.32 ± 0.01	0.18 ± 0.00	24.54 ± 0.93	7.29 ± 0.20
A4	0.30 ± 0.04	0.52 ± 0.06	16.27 ± 0.57	7.98 ± 0.17
A5	0.50 ± 0.01	0.82 ± 0.01	24.01 ± 0.76	14.44 ± 0.65
A6	0.31 ± 0.00	2.46 ± 0.04	26.57 ± 0.46	15.77 ± 0.25
A7	0.58 ± 0.01	0.32 ± 0.00	20.55 ± 0.67	3.85 ± 0.08
A8	0.43 ± 0.01	0.10 ± 0.00	24.97 ± 0.58	7.83 ± 0.25
A9	0.60 ± 0.01	2.38 ± 0.05	19.71 ± 0.23	15.87 ± 0.15
A10	0.23 ± 0.00	3.27 ± 0.04	23.51 ± 0.55	14.59 ± 0.31
B1	0.02 ± 0.00	0.49 ± 0.01	2.54 ± 0.04	2.12 ± 0.03
B2	0.09 ± 0.00	0.36 ± 0.00	1.85 ± 0.03	0.35 ± 0.00
B3	0.28 ± 0.00	0.45 ± 0.01	2.63 ± 0.04	3.11 ± 0.04
B4	0.15 ± 0.01	0.50 ± 0.01	1.97 ± 0.05	2.59 ± 0.04
B5	0.90 ± 0.01	0.85 ± 0.03	10.42 ± 0.37	2.51 ± 0.04
C1	0.31 ± 0.01	0.56 ± 0.02	2.05 ± 0.03	3.49 ± 0.04
C2	0.02 ± 0.01	1.13 ± 0.02	5.94 ± 0.10	3.92 ± 0.04
C3	1.31 ± 0.04	0.32 ± 0.01	2.55 ± 0.06	0.47 ± 0.01
C4	0.47 ± 0.01	1.31 ± 0.04	11.71 ± 0.41	4.92 ± 0.09
C5	0.39 ± 0.01	0.67 ± 0.02	8.21 ± 0.10	2.62 ± 0.09

## 3. Experimental

### 3.1. Reagents and Chemicals

HPLC-grade acetonitrile, methanol and formic acid were purchased from Sigma Aldrich (St. Louis, MO, USA). Ultrapure Water (18.2 MΩ) was produced by Milli-Q water system (Millipore, Bedford, MA, USA). Twenty samples of roots of CPM and PPM were collected from various habitats in China, and authenticated by Professor Zhihai Huang in our lab. The related information is summarized in the Supplementary Data (Table S1). The standards of gallic acid (GA), emodin (EM) and *trans*-2,3,5,4′-tetrahydroxystilbene-2-*O*-β-d-glucopyranoside (THSG) were obtained from the National Institutes for Food and Drug Control (Beijing, China). Emodin-8-*O-*β-d-glucopyranoside (EMG; over 98% purity by HPLC) was isolated in our laboratory.

### 3.2. Chromatography and MS Conditions

LC analyses were performed on a Thermo Accela UHPLC system (Thermo Fisher Scientific, San Jose, CA, USA) equipped with a quaternary pump, a diode-array detector (DAD), an auto-sampler, and a thermostatically column compartment.

After also running optimization analyses on both Hypersil C_18_ (2.1 mm × 100 mm, 5 μm) and Kinetex XB C_18_ (2.1 mm × 100 mm, 1.7 μm) columns, we found the best overall resolution on an AcQuity UPLC™ BEH C_18_ column (2.1 mm × 50 mm, 1.7 μm) at room temperature (see Section 2.1) and used this column for all subsequent runs. The mobile phase was also optimized by comparing an acetonitrile-H_2_O mobile to a methanol-H_2_O system and with the addition of various modifiers to the acetonitrile-H_2_O mobile, including formic acid, acetic acid, ammonium acetate, and ammonium formate, all at 0.1% (see Section 2.1). The final mobile phase was composed of acetonitrile (A) and water containing 0.1% formic acid (B) using the following gradient program: 13% A (0 min), 35% A (3.5 min), 90% A (7.5 min), 95% A (8.5 min) and 95% A (10 min). A pre-equilibration period of 4 min was used between individual runs. The mobile phase flow rate was 400 μL/min, and the injection volume was 2 μL. The online UV spectra were recorded in the range of 200–400 nm.

Mass spectra were acquired using a Thermo-Fisher LTQ-Orbitrap XL hybrid mass spectrometer, which was connected to LC system via an electrospray ionization (ESI) source as interface. The basic conditions of MS analysis were as follows: the mass spectrometer parameters were negative ion mode, ion spray voltage at 3500 V, capillary voltage at 37 V, capillary temperature at 300 °C, sheath gas flow rate at 40 psi and auxiliary gas flow rate at 4 psi. The scan spectra were from *m*/*z* 150 to 1200.

For qualitative analysis of CPM and PPM, the Orbitrap resolution of survey scan was set as 30000 and MS^n^ scan was set 15000. The data-dependent MS^n^ scanning was performed to trigger fragmentation spectra of target ions and to prevent repetition by dynamic exclusion settings. For quantitative determinations of four compounds, the MS detection was operated in linear ion trap (LTQ) with selected reaction monitoring (SRM). The ion trap collision induced dissociation (CID) mode was used for SRM fragmentation and the ions transitions are as follows: GA (*m*/*z* 169→125), EM (*m*/*z* 269→225), THSG (*m*/*z* 405→243) and EMG (*m*/*z* 431→269). The selected ion width was *m/z* ±1 and the normalized collision energy was set 35%.

### 3.3. Sample Preparation

The dried roots were powdered to a homogeneous size by a mill, sieved through a No.60 mesh (250 μm), and further dried at 50 °C in the oven for 6 h to constant weight. The powdered sample accurately weighed 0.2 g was extracted with 25 mL of methanol in a round-bottomed flask and the mixture was heated under reflux for 0.5 h at 70–75 °C, and cooled at room temperature. Methanol was added to compensate for the lost weight. The extracted solution was centrifuged at 12,000 rpm for 10 min, after filtration through a 0.45 μm membrane, an aliquot of 10 μL of the filtrate was injected into the UHPLC-MS system for LC-MS analysis.

### 3.4. Preparation of Standard Solutions

The reference standards were accurately weighed and dissolved in methanol to prepare stock solutions. All standards were completely dissolved in the mixed standard working solution of 8 μg/mL for gallic acid, 2.75 μg/mL for EM, 210 μg/mL for THSG, 228 μg/mL for EMG, respectively. For the construction of calibration plots, the standard stock solution was further diluted with methanol to make seven different concentrations at 1/2, 1/4, 1/8, 1/16, 1/32, 1/64, and 1/128 of the working solutions. All solutions were stored in a refrigerator at 4 °C for analysis.

### 3.5. Statistical Analysis

The mass data acquired were imported into the Xcalibur software (version 2.1) (Thermo Fisher Scientific, CA, USA) for peak detection and alignment. Prism 5.0 was used to run two-way ANOVAs with Bonferroni corrections on ion peak areas of twelve investigated components (sucrose, gallic acid, procyanidin B, catechin, 6-methoxyl-2-acetyl-3-methyl-juglone-8-*O*-glu, tetrahydroxy stilbene-*O*-di-glu, THSG, citreorosein-*O*-glu, torachrysone-*O*-glu, emodin-8-*O*-glu, emodin-*O*-(malonyl)-glu and emodin) to test for differences between PM and its processed products. When the peak areas of investigated compounds were difficult to integrate or not detected in the samples, the values of such data was considered to be zero.

## 4. Conclusions

In this study, a simple, rapid and accurate UHPLC-MS method was established to qualitatively and quantitatively determine the major components of PM and PPM. The method was used to successfully quantify four components in twenty batches of PM and PPM samples. This novel approach is a highly useful technique to identify constituents and control the quality of CPM and PPM, it also offers incredible advantages, including speed, simplicity, and a reduction in solvent consumption. The method would become an important quality control technique for Chinese medicine and could be adopted widely.

## Supplementary Materials

Supplementary materials can be accessed at: http://www.mdpi.com/1420-3049/20/12/19813/s1.
