# Supplementary material for: Application of Ultra-High-Performance Liquid Chromatography Coupled with LTQ-Orbitrap Mass Spectrometry for the Qualitative and Quantitative Analysis of Polygonum multiflorum Thumb. and Its Processed Products
Source: Molecules. 2015 Dec 26;21(1):40. doi: 10.3390/molecules21010040 (PMC6272829; doi:10.3390/molecules21010040)
Supplement: Supplementary file 1 [file molecules-21-00040-s001.pdf]

Supporting Information: Application of Ultra-High-Performance Liquid Chromatography Coupled with LTQ-Orbitrap Mass Spectrometry for the Qualitative and Quantitative Analysis of *Polygonum multiflorum* Thumb. and Its Processed Products

Teng-Hua Wang, Jing Zhang, Xiao-Hui Qiu, Jun-Qi Bai, You-Heng Gao and Wen Xu

Table S1. Crude drug and its processed products collected in the present study.

Sample No.	Processing Methods	Origins	Batch No.
A1	Crude	Deqing County, Guangdong Province	20090527
A2	Crude	Guiming County	Y91619
A3	Crude	Deqing County, Guangdong Province	080817
A4	Crude	Gaozhou County, Guangdong Province	20080801
A5	Crude	Sichuan Province	Y090618
A6	Crude	Sichuan Province	Y090624
A7	Crude	Gaozhou County, Guangdong Province	20080801
A8	Crude	Deqing County, Guangdong Province	090817
A9	Crude	Sichuan Province	Y090620
A10	Crude	Sichuan Province	Y090621
B1	Black soybean processing	Gaozhou County, Guangdong Province	2008080124ht
B2	Black soybean processing	Gaozhou County, Guangdong Province	200808012ht
B3	Black soybean processing	Gaozhou County, Guangdong Province	2008080124hh
B4	Black soybean processing	Gaozhou County, Guangdong Province	200808012hh
B5	Black soybean processing	Deqing County, Guangdong Province	20080817h
C1	Steaming processing	Guangdong Province	100900531
C2	Steaming processing	Sichuan Province	090723
C3	Steaming processing	Guizhou Province	090720
C4	Steaming processing	Sichuan Province	090722
C5	Steaming processing	Guangdong Province	090721
